# A Commercial Arbuscular Mycorrhizal Inoculum Alleviated the Effects of Acid Water on *Lupinus angustifolius* Grown in a Sterilized Mining Dump

**DOI:** 10.3390/plants12101983

**Published:** 2023-05-15

**Authors:** Aurora Neagoe, Virgil Iordache

**Affiliations:** 1 “Dan Manoleli” Research Centre for Ecological Services—CESEC and “Dimitrie Brândză” Botanical Garden, University of Bucharest, Aleea Portocalelor No. 1-3, Sector 6, 060101 Bucharest, Romania; 2 Department of Systems Ecology and Sustainability, and “Dan Manoleli” Research Centre for Ecological Services—CESEC, University of Bucharest, Spl Independentei 91-95, Sector 5, 050089 Bucharest, Romania

**Keywords:** non-host plant, commercial AMF inoculum, phosphorus, heavy metals, phytoremediation, soil acidification, oxidative stress

## Abstract

*Lupinus* species have been sporadically reported to be colonized by arbuscular mycorrhizal fungi (AMF). The interactions between AMF and lupine plants could also be non-symbiotic, from positive to negative, as controlled by the stress conditions of the plant. The goal of the study was to reveal the existence of such positive interactions and provide preliminary data for a myco-phytoremediation technology of mining dumps using *L. angustifolius* as a first crop. The objective was to test the hypothesis that the AMF inoculation of an acidified dump material contaminated with heavy metals would improve the growth of *L. angustifolius* and decrease oxidative stress. The design consisted of a one-month bivariate pot experiment with plants grown in a mining dump soil inoculated and not inoculated with a commercial AMF inoculum sequestered in expanded clay and watered with acidic and neutral water. There was no AMF root colonization under the experimental conditions, but under neutral and acidic water conditions, the phosphorus concentrations in roots and leaves increased, and the superoxide dismutase and peroxidase activities significantly decreased due to AMF inoculation. The increase in leaf phosphorus concentration was correlated with the decrease in peroxidase activity. The fresh weight of shoots and leaves significantly increased due to the commercial inoculum (under acidic water conditions). At the end of the experiment, the ammonium concentration in the substrate was higher in the inoculated treatments than in the not inoculated ones, and the concentrations of many elements in the dump material decreased compared to the start of the experiment. A comprehensive discussion of the potential mechanisms underlying the effects of the commercial AMF inoculum on the non-host *L. angustifolius* is completed.

## 1. Introduction

Although old studies found no root colonization with arbuscular mycorrhizal fungi (AMF) of plants from *Lupinus* genus [1,2], a more recent review pointed out that mycorrhizal fungi can colonize *Lupinus* species at low rates [3]. The symbiotic status depends on lupine species, fungal taxa, and growth conditions [3]. The nature of the relations needs to be understood by detailed field surveys and experiments. The interactions between AMF and lupine plants can also be non-symbiotic. Non-mycotrophic plant species can stimulate the germination and hyphal growth of AMF [4] at time scales of 3 to 4 weeks, which may be enough to change soil conditions and influence plant development. The interactions can vary on a continuum range [5] from positive to negative and are controlled by the stress conditions of the plant.

Lupine species are often used to stabilize soils contaminated with heavy metals (HMs) [6]. Previous studies have demonstrated the usefulness of *Lupinus luteus* inoculation with plant growth-promoting bacteria in metal-polluted soils [7,8], but investigations on the potentially positive role of AMF inoculation are lacking. Furthermore, acid runoff conditions may occur in areas contaminated with HMs [9], providing even more stress for plant growth in the HMs substrate. The acidity may originate from the soil organic material [9], from sulfide-bearing rocks [10], acid sulfate soil [11], or tailings [12]. They all lead to continuous or intermittent stream acid events [13]. Another source of acid water can be acid rain, especially in some geographic regions [14]. The acidity of the water entering the system by runoff, flooding, or precipitation [15] adds supplementary stress to the plants growing in contaminated soils [16].

Some lupine species are more adapted to acid soil conditions than others [17]. *L. angustifolius* is not well suited to strongly acid ones, while *L. luteus* has a high tolerance to soil acidity [18].

In addition to the direct effect of soil acidity on plants, the acid water also changes the phytoavailability of HMs in soil [19] and other chemical and enzyme activities [20,21]. This increases the toxic effects on plants in a complex synergic way [22].

Plant seedlings inoculated with mycorrhizal fungi have been reported to better tolerate such complex stress [23]. AMF enhanced in some cases nutrient acquisition and reduced aluminium toxicity under acid rain [24], enhanced photosynthetic parameters [25], and decreased superoxide dismutase activities [26]. However, older studies reported that other plant species did not benefit from AMF under acid rain conditions [27]. The colonization by extraradical AMF mycelium decreased the antioxidant enzymatic activity [28,29], but in other cases, their activities were increased [30]. The fungi themselves have a different tolerance to acid water conditions [31,32]. They respond to acid stress by physiological adaptation [33] and habitat segregation along pH gradients [34]. Overall, AMFs are reported to alleviate the acid soil stress in plants [35,36].

Thus, there are basic science knowledge gaps related to the potential positive effects of AMF on lupine resistance to the stress due to acid water in mining waste environments. However, the significance of this work is mostly related to applied science gaps in the field of myco-phytoremediation of mining dumps. As lupine is a good first plant when using crop rotation to accelerate secondary succession during remediation [37], we explored the possibility to mix a commercial AMF inoculum with the mining dump before the start of the lupine first crop. The mycorrhizal fungi would be able to colonize a second host crop if they would survive during the first crop by interactions with the non-host lupine in the high stress conditions. The goal of the study was to reveal the existence of such positive interactions and provide preliminary data for a myco-phytoremediation technology of mining dumps using *L. angustifolius* as a first crop. The pot experiment reported here was part of a larger set of treatments, including other contaminated substrates from mining areas, and coupled with a lysimeter experiment, all testing hypotheses about the effect of a commercial AMF inoculum on *L. angustifolius* whose results will be presented in other manuscripts (Figure S1).

Accordingly, the objective of this study was to test the hypothesis that the AMF inoculation of a dump material under neutral and acid water conditions would improve the growth of *L. angustifolius* and decrease oxidative stress. The estimated parameters were selected in line with previous work in mining areas, which demonstrated their efficiency and effectiveness in monitoring the plant development during phytoremediation of mining areas [38,39,40]. 

## 2. Materials and Methods

To test the hypothesis, a bivariate pot experiment with *L. angustifolius* grown in a mining dump soil inoculated and not inoculated with AMF and watered with acid and neutral water was performed. We added two treatments with *L. angustifolius* growing on not contaminated soil to have a reference for a general comparison of results. 

### 2.1. Soils and Water Characterization

The dump material used for this experiment (brown podzolic, according to [41]) was sampled from the Gessenhalde uranium dump Sorge Settendorf, located in the Ronneburg mining area at ≈6 km SW of Seelingstädt (50°44′0″ N, 12°13′0″ E), Thuringia, Germany. This soil was sampled from the upper 20 cm in 20 subsamples to cover the heterogeneity of the contaminated area. The subsamples were manually homogenized and brought together into one composite sample. The reference soil was sampled from Miesitz (50°44′0″ N, 11°50′0″ E), Saale-Orla Kreis, Thuringia, Germany. These two soils were analyzed from a physicochemical point of view, and the results of the measured variables are presented in Table 1. Two types of water were used to irrigate these soils, namely, neutral distilled water and acidic water prepared in the laboratory by diluting a concentrated hydrochloric acid (HCl 37% p.a. Merck) at a concentration of 10^−3^ M. The mine dump soil already had an acidic pH (4.88), so we needed slightly acidic water to be able to lower the pH of the mining substrate slightly. We did germination tests in the laboratory where we used three different concentrations of acidic water for irrigation, namely, 0.1 M, 0.01 M, and 0.001 M. The one that allowed the plant to develop was acidic water with a concentration of 0.001 M.

### 2.2. Experimental Setting

The two homogeneous composite soils were sterilized (2.2 bar, 121 °C, 30 min × 2 times) before being used for filling each 400 mL polyethylene pot. Six experimental treatments with four replicates each were prepared, as can be seen in Table 2. Reference (R) and dump material soils were used, inoculated (DF) or not (D), with arbuscular mycorrhizal fungi (AMF) *Glomus intraradices* species (current name *Rhizophagus irregularis*) sequestered in expanded clay, provided by Institute für Pflanzenschutz, Hannover University, Germany. The purchased product AMF 510 contained 115 spores/g substrate, and 10% was used in each pot [42]. The quality of the AMF inoculum is in accordance with the Committee of Mycorrhiza Application Germany (CMAG, 12/1997, [42]). Using expanded clay is a generalized practice for the preservation and transport of AMF. The addition of commercial inoculum slightly changed the chemical variables of the dump substrate because of the expanded clay (Table 1).

The soils for each treatment were carefully fertilized to avoid the inhibition of mycorrhizal symbiosis [43], using 0.1 g per pot, as NH_4_NO_3_ × 2 times (at the time of sowing and after 15 days) and watered with 60 mL distilled water per pot at the time of installation. Irrigation was completed twice a day to maintain the soil moisture content, with neutral or acidic water, depending on the type of treatment. After preliminary germination tests, six seeds of *Lupinus angustifolius* L. cv Bordako per pot were sown. The experiment was carried out for one month (as the lupine plants grow fast) in a growth chamber under fully controlled conditions: 70% relative humidity and a light/dark cycle regime of 16 h light/22 °C and eight hours night/16 °C with Sylvania cool-white incandescent lamps, 11 W m^−2^.

### 2.3. Measurement of Soil Variables

Before starting and at the end of the experiment, soil variables were measured: soil moisture by drying the samples at 105 °C until constant weight, soil solution pH (using distilled H_2_O) (WTW 320, Weilheim, Germany), loss on ignition (LOI) by the disintegration of organic material in an oven at 600 °C, and concentration of nitrate and ammonia. For these two last variables, soil samples were stored at 4 °C and processed within 24 h of sampling. For nitrate and ammonia, we weighed 20 g from each sample. It was extracted using 100 mL of 0.2 M KCl solution, filtered through glass filters (Whatman GF/C) and spectrophotometrically analyzed by sodium nitroprusside method for ammonia and by sulfosalicylic acid method for nitrate. Air-dried soil samples were sifted through <2 cm mesh size sieve and digested using aqua regia [44]. Then, the concentration of elements was analyzed using an ICP-AES (Liberty 150, Varian, Palo Alto, CA, USA). All methods are described in detail in [39].

### 2.4. Measurement of Plant Variables

After harvesting, the plants were washed with abundant tap water, rinsed several times with distilled water, partitioned into roots and aboveground parts, and weighed for fresh weight (f.w.) determination. For histochemical staining 10 root fragments from each pot were preserved in a solution prepared from ultrapure water, 45.85% (*v*/*v*) ethanol, 6% (*v*/*v*) formaldehyde, and 2.3% (*v*/*v*) acetic acid, according to Schmitz et al. (1991). Then, all the aboveground and underground biomass was frozen until processing. All plant material was lyophilized (Martin Christ, Osterode am Harz, Germany) for determination of dry weight (d.w.), ground in a stainless-steel mill that was carefully cleaned between samples (IKA, All basic, Wilmington, NC, USA), and stored at −20 °C. All ground plant samples were wet digested (0.2 g of plant material and 2 mL of suprapur Merck HNO_3_) using microwave-assisted pressure (Mars 5, CEM, Kamp-Lintfort, Germany). The quality assurance and control criteria were met by checking the standard reference material CRM 281 for ryegrass. The differences were not more than 5%. All elements were measured using the same instruments as described by the measurement of soil variables.

Furthermore, non-enzymatic and enzymatic variables were assessed. Thus, the photosynthetic pigments, such as chlorophyll a and b, and carotenoids, were determined by homogenizing 30 mg (d.w.) aboveground plant material in acetone solution (80% acetone: 15% water: 5% NH_3_-solution [25%], *v*/*v*) at 75,000 rpm for 30 s, using an ultraturax Heidolph Scilent Crusher S. All homogenized samples were centrifuged at 4800 rpm for 20 min at 4 °C. The obtained supernatant was then spectrophotometrically measured at 480, 645, 647, 663, and 644 nm [45].

For total protein extraction and determination, 100 mg plant materials were used (roots and aboveground part) homogenized in 4 mL cold potassium phosphate buffer (100 mM, at pH 7.2) in which was added 2% polyvinylpyrrolidone, 2 mM chelaplex III (EDTA), and 2 mM dithioerythritol (DTE). The homogenized samples were, after that, centrifugated for 20 min at 6000 rpm and 4 °C, and the supernatant was dialyzed using a potassium phosphate buffer (5 mM, at pH 7.2) at 4 °C, for 12 h. For a brief description of the method, we specified that the dialyzed supernatant was first precipitated with 10% trichloric acid, then solubilized with 1N NaOH. As a standard for performing the calibration curve bovine serum albumin (BSA) was used, then the supernatant was further used to determine protein concentration [46].

Enzyme assays, such as superoxide dismutase (SOD) and peroxidase (POD) activity, were performed. SOD activity was assessed by ferricytochrome c (prepared in 50 mM sodium phosphate buffer: pH 7.8) method using xanthine (2,6 Dihydroxypurine SIGMA) /xanthine oxidase (XOD, 40 units/4.2 mL, SIGMA) as the source of superoxide anions (O_2_^−^). One unit of activity was defined as the amount of enzyme necessary to inhibit the reduction in cytochrome c in a ratio of 50% [47]. POD activity was assessed after the reaction of protein extract with guaiacol, and hydrogen peroxide (30%) dissolved in 50 mM citric acid/sodium phosphate buffer (pH 5). In the presence of H_2_O_2_, POD catalyzes the transformation of guaiacol to tetraguaiacol. The measurement of POD was performed at 470 nm and 30 °C with guaiacol [48]. 

Lipid peroxides (LP) were measured in terms of the malondialdehyde (MDA) content obtained by decomposing the peroxides of polyunsaturated fatty acids. A total of 100 mg (d.w.) of the sample was homogenized with 4 mL of thiobarbituric acid (TBA) solution at 75,000 rpm for 30 s. After that, samples were centrifugated at 6000 rpm for 15 min at 4 °C. The resulting supernatant was finally spectrophotometrically measured at 440, 532, and 600 nm (according to [49]). 

All methods were described in detail by [39,50].

### 2.5. Root Mycorrhizal Colonization

All fragments of roots preserved in the fixing solution, as described at the beginning of the measurement plant variables section, were further treated with 2% (*w*/*v*) KOH and placed in a water bath at a temperature of 95 °C for 15 min twice. They were then rinsed with distilled water three times and incubated with 2% (*v*/*v*) HCl for 30 min, then stained for 90 min with microscopy lactophenol blue solution for staining fungi (Merck, Darmstadt, Germany). Finally, they were washed and preserved (at 4 °C) using 50% lactic acid [51]. All root fragments stained as described were transferred to slides using 100% glycerol and microscopically visualized by Carl Zeiss Axio Imager 2 microscopy (Jena, Germany) at ×40 magnification. There was no mycorrhization under the described experimental conditions (neither vesicles nor arbuscles were observed).

### 2.6. Data Processing

Cd, Co, Pb, and V concentrations in soils and plants have not been included in the analysis because of the many under-detected values in plants (the values of the concentrations of these elements can be found in the raw data set available in Supplementary Materials). A two-way ANOVA was performed for the core experimental treatments (dump soil with or without AMF, with neutral or acid water) and another one to compare with the reference soil conditions (dump or reference soil, with neutral or acid water). We could not perform a three-way ANOVA because we did not have treatments of reference soil with AMF (for reasons of costs and because we assumed that the effect of AMF on the non-host plant would be detectable only under stress conditions). After visual inspection of the plots of differences between averages, a one-way ANOVA was also performed in some cases when the effect of inoculation differed greatly between variants with neutral and acid water. For visual simplicity, we also represented the results of a two-way ANOVA with three soil treatments (reference, dump material not inoculated, and dump material inoculated). Still, the statistical significance indicated in the figures is extracted from the separate ANOVA described above. To have an overall image of the correlations between measured variables and the location of the experimental treatments in the multidimensional space, we performed a principal component analysis. In addition to the biplots of the scores of variables on the extracted factors, revealing the correlations between variables, we also plotted the scores of each experimental replicate (pot) on the first and second extracted factors. In this way, the clustered distribution of the experimental treatments and their relative locations on the range from minimum to maximum values of the variables is revealed. For data processing, we used Statistica version 14.0.0.15 (Tibco Software Inc., Palo Alto, CA, USA), and for the figures, the same software and Excel 2019.

## 3. Results

The raw data set is available following the link provided in the Supplementary Materials. We present the results following the causal chain from concentrations of elements in plants to oxidative stress variables and to plant biomass. Then, we compare the soil variables at the end of the experiment with those at the start of the experiment.

### 3.1. Phosphorus, Potassium, and Other Elements

**Figure 1:**
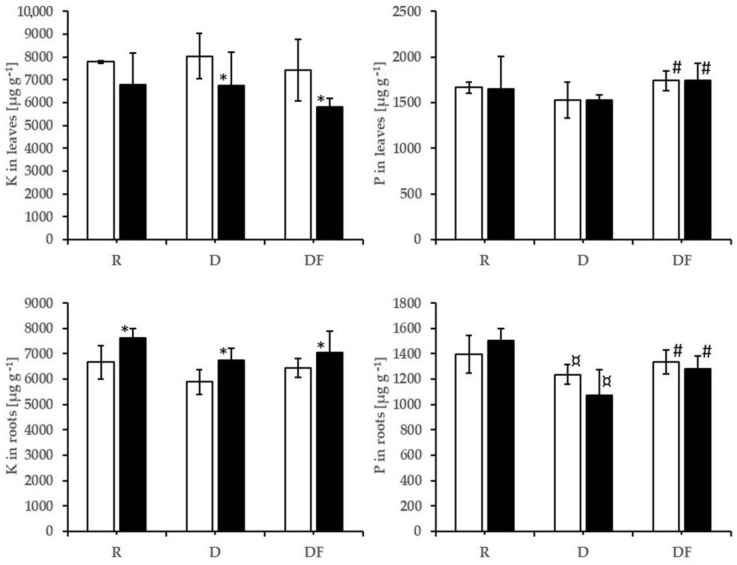
Phosphorus and potassium concentrations in roots (**down**) and leaves (**up**). The treatments with neutral water are represented by white columns, and in black are those with acid water. The black stars (*) indicate that the differences due to the acid water on R, D, or DF substrates are statistically significant (ANOVA). The symbol ¤ indicates that the differences between D and R variants are statistically significant. The symbol # indicates that the addition of commercial AMF inoculum to the D substrate led to a significant increase in P concentrations in roots and leaves.

There are differences between many elements in plants’ roots and leaves from reference soil and those in the dump material (Tables S1 and S2). Ca, P, and Sr concentrations in roots decreased, while Cu, Fe, Mg, Mn, Ni, and Zn concentrations increased. Sr concentrations in leaves decreased, and Ca, Cu, Fe, Mg, Ni, and Zn increased. The use of acid water for not inoculated substrates increased the Ca concentrations in roots, decreased the K concentration (Figure 1, Table S1), and had no effect on the elements in leaves.

The inoculation significantly increased the P concentration in roots and leaves, both in the treatments with distilled—neutral and with acid water (Figure 1)—and led to an increase in the concentrations of Sr and Zn in roots. The acid water on the inoculated variants increased the Ca, K, and Sr concentrations in roots, increased the Ca and Mn concentrations in leaves, and decreased the K concentrations in leaves (Figure 1, Tables S1 and S2).

### 3.2. SOD, POD, LP, Protein, Assimilating Pigments

**Figure 2:**
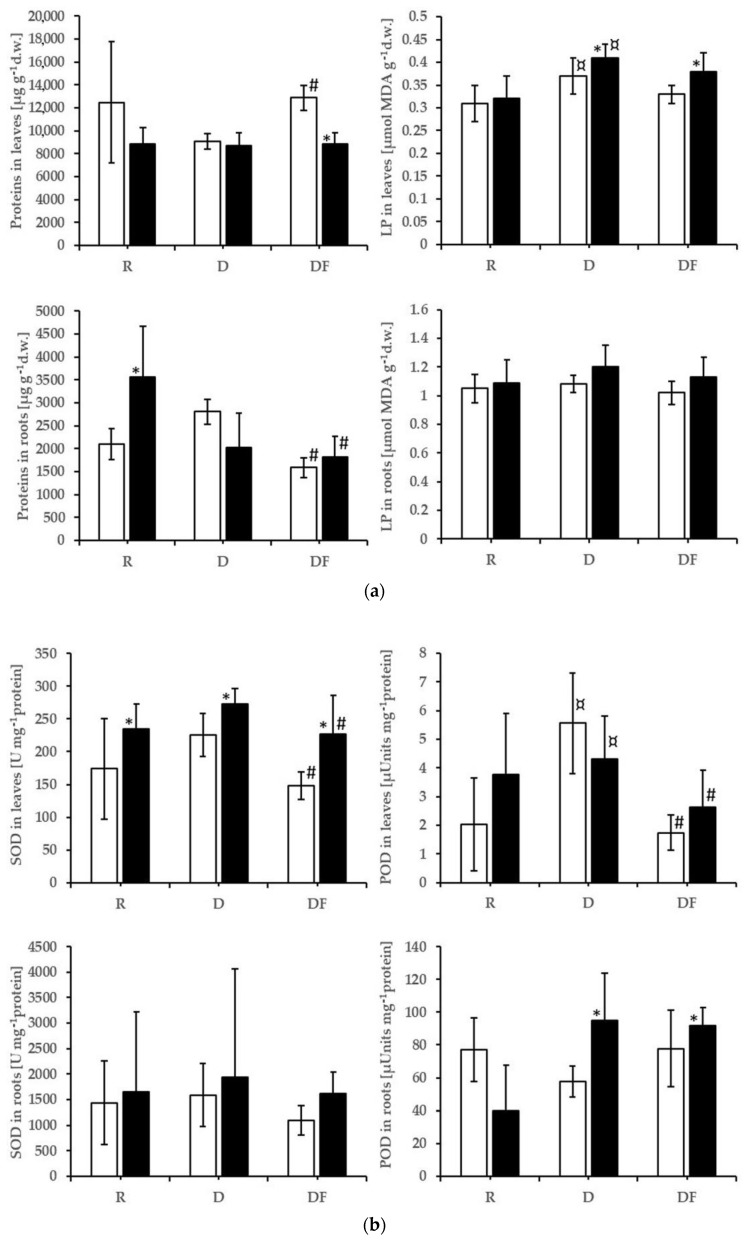
(**a**) Protein concentrations and LP in roots (down) and leaves (up). The treatments with neutral water are represented by white columns, and in black are those with acid water. The black stars (*) indicate that the differences due to the acid water on R, D, or DF substrates are statistically significant (ANOVA). The symbol ¤ indicates that the differences between D and R variants are statistically significant. The symbol # indicates that the average value of a variable in DF substrate is different from its average value in D. (**b**) SOD and POD activities in roots (down) and leaves (up). The treatments with neutral water are represented by white columns, and in black are those with acid water. The black stars (*) indicate that the differences due to the acid water on R, D, or DF substrates are statistically significant (ANOVA). The symbol ¤ indicates that the differences between D and R variants are statistically significant. The symbol # indicates that the addition of commercial AMF inoculum to the D substrate caused a significant decrease in SOD and POD in leaves.

The POD and LP in leaves significantly increased from the reference soil to the dump material (Figure 2a,b). The concentrations of chlorophyll and carotenoids decreased (Table S3). The acid water significantly increased the SOD activity (Figure 2b) and significantly reduced the concentrations of carotenoids (Table S3), both in the plants grown on reference soil and dump material. The protein concentration in leaves decreased in treatments with acid water and increased when the dump material was inoculated (Figure 2a). SOD and POD activities decreased in leaves as a result of inoculation (Figure 2b) and the carotenoids concentrations (Table S3). In roots, the protein concentration was decreased by inoculation, and the POD activity increased in the dump material with acid water compared with dump material with neutral water. Averages, standard deviations, and results of ANOVA are available for these variables in Tables S3 and S4. The oxidative stress variables in stems have been measured on composite samples because the amounts of dry biomass in each pot were too small. In the raw data set, one can notice that the values of oxidative stress variables in composite samples of stems have the same pattern of decrease in the inoculated treatments, but ANOVA tests could not be performed. 

### 3.3. Plant Biomass

**Figure 3:**
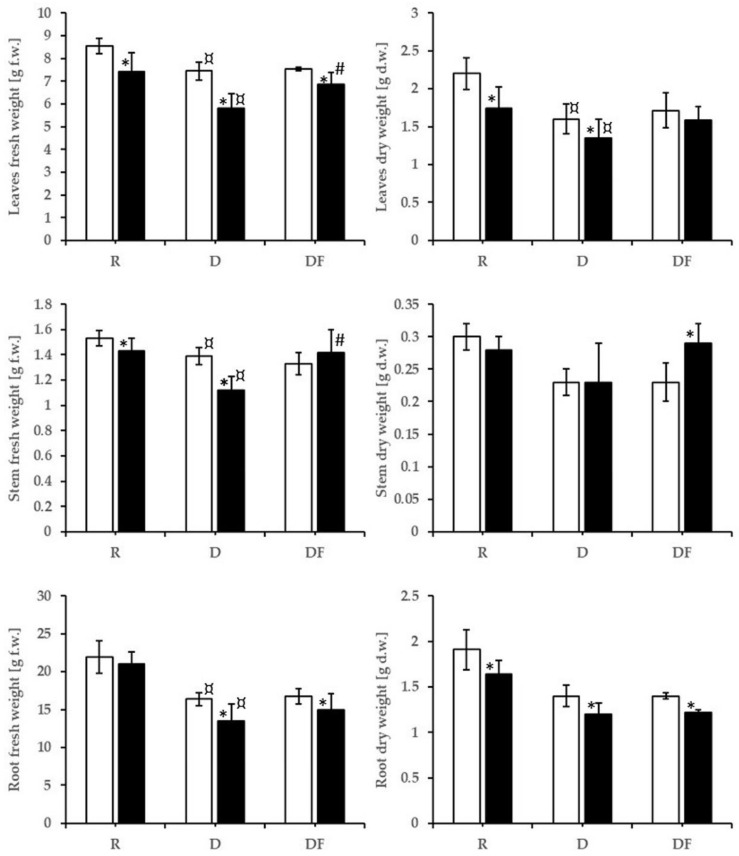
Fresh weight and dry weight of roots (**down**), stems (**middle**), and leaves (**up**). The treatments with neutral water are represented by white columns, and in black are those with acid water. The black stars (*) indicate that the differences due to the acid water on R, D, or DF substrates are statistically significant (ANOVA). The symbol ¤ indicates that the differences between D and R variants are statistically significant. The symbol # indicates that the average value of a variable in the DF substrate is different from its average value in D.

The fresh weight and dry weight of roots significantly decreased from the reference soil to the dump material, and the acid water further reduced the dry weight of roots in both treatments. The results of the ANOVA test showed the differences between treatments (Tables S3 and S4). The height, fresh weight, and dry weight of stems were smaller when the plants grew on the dump substrate compared to the reference soil, and the stems’ fresh weight was further decreased by acid water. A similar decrease in biomass due to contamination and acid water occurred for leaves. One can now look at the effect of inoculation with AMF (Figure 3, Tables S3 and S4). The fresh weight of plants’ stems and leaves in acid water treatments increased due to soil inoculation, while the height of the plant’s shoots decreased. No significant effect of AMF on the dry weight was observed (the average increased at a *p* level 0.17).

### 3.4. Principal Component Analysis of Plant Variables

**Figure 4:**
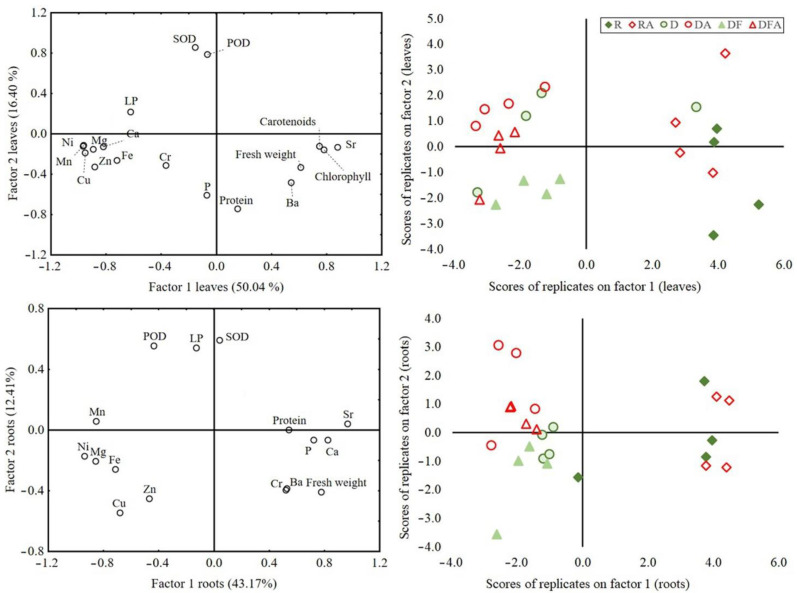
(**Left**) Biplots resulted from principal component analysis of plant variables in roots (**down left**) and leaves (**up left**), representing correlations between variables and the extracted factors. (**Right**) Scatterplots of loadings of all samples (pots) on factors 1 and 2 extracted by PCA. R, D, and DF in the (**up right**) legend stand for the treatments with neutral water, and RA, DA, and DFA for those with acid water.

Preliminary correlation matrixes revealed the significantly correlated variables in roots and leaves (Table S5). The two significant extracted factors accounted for 55.58% of the variability in roots and 66.44% in leaves (Figure 4). Root oxidative stress variables were negatively correlated with P concentration and fresh-weight biomass. In leaves, the same pattern holds for superoxide dismutase and peroxidase activities. Lipid peroxidation in leaves was positively correlated with the concentrations of a cluster of elements mobilized from the dump material (Ni, Cu, Zn, and Fe) and negatively correlated with leaves’ fresh weight, concentrations of assimilating pigments, and protein concentration. From the perspective of the experimental treatments, one can notice the clear separation between the location of the sample loadings in the multivariate space, confirming the ANOVA results. The effect of acid water is more evident in the case of the dump material and more prominent in roots than in leaves. The inoculation with AMF visibly separated the samples (pots) based on the leaf variables.

A correlation matrix analysis restricted to leaf variables of plants grown in dump material (n = 16, not inserted, raw data are provided in the Supplementary Materials for reconstruction) demonstrated a significant negative correlation between peroxidase activity and the concentrations of P (Figure S2, R = −0.63, *p* = 0.009). The visual separation of the experimental treatments in Figure S1 corresponds to the statistically significant differences proved for these variables with ANOVA.

### 3.5. Substrate Variables at the End of the Experiment

Nitrate and ammonium in soil increased significantly from the beginning to the end of the experiment. There were many cases of a significant decrease in element concentrations in the substrate at the end of the experiment (Table 3).

Table S6 includes averages, standard deviations, and results of ANOVA for soil variables at the harvesting time. The acid water significantly decreased the pH and increased the nitrate concentration in the substrate both in the reference soil and the dump material. The inoculation increased the ammonium concentration in the dump material under neutral and acid water conditions.

In order to inspect if the changes in dump material chemistry as a result of expanded clay addition by the commercial AMF inoculum at the start of the experiment influenced the chemical variables of substrate and plants at the end of the experiment, we compared the corresponding trends (Table 4). One can see that the trends for Al, K (increase), and Ni and Mg (decrease) in the substrate are similar at the end compared with the start of the experiment (Table 4). This did not occur for Zn, whose concentration in the substrate of the pots strongly decreased by the end of the experiment. When one looks at the concentrations of these elements in roots and leaves, one can see that they mirror the pattern found in the substrate. The addition of the commercial AMF inoculum did not change Al, Mg, K, or Ni in plants but only increased the concentrations of P, Sr, and Zn.

## 4. Discussions

### 4.1. Effects of Independent Variables: Substrate Type, Acid Water, and Commercial Inoculum

We used experimental treatments with reference soil for general comparison, and the values of variables of plants grown in D treatment compared with those in R treatment followed the expected patterns. The P concentration in D plants is smaller than in R plants, the concentrations of many elements are larger, and the SOD increased in D plants. As a result, the fresh weight of all plant parts in D treatment, and the dry weight of leaves, are smaller than in R treatment.

*L. angustifolius* has been previously used for mine tailing’s revegetation [52]. An option to stimulate the *L. angusifolius* development on dump material conditions is the use of an amendment, such as compost [39].

Blue lupine can also be used as a pre-preceding leguminous crop [53]. For instance, Zhong and colleagues found that *L. angustifolius* eco-engineered the mine tailings by enhancing the N status of tailings and mobilizing primary mineral P into organic P [54]. 

Past work with simulated acid water demonstrated that the effects depend on the particular soil-plant-microbial conditions and on the chemical properties of the acid water. As it is difficult to disentangle the separate effect of each variable in experiments with acid water, one can expect that the observed patterns will be specific to each ecological context [55]. Plant diseases due to soil microorganisms can be inhibited by acid water, as demonstrated by *L. angustifolius* [56]. However, the disease response to the acidity of simulated rain was system-dependent [57]. In another case, the number of fungal propagules in the rhizosphere tended to be increased by acid water [58]. The plant growth and all microbial populations were even stimulated when the acid water penetrated the soil [59]. The overall effects of acid water on plants development are species-specific and involve always restructuring of the soil microbial communities [60].

In our experimental conditions, the effects of acid water on the development of *L. angustifolius* at a scale of one month were negative. This was indicated by the increase in oxidative stress variables (LP, SOD, POD) in all treatments and the decrease in the roots’ dry weight and the leaves’ fresh weight in all treatments. The use of acid water instead of neutral water added extra stress to plant development, besides the mining dump conditions.

As we did not observe colonization of the inoculated plants with AM fungus, it cannot be argued that the observed effects from inoculation were caused by this particular fungus. The inoculum could also contain bacteria or other fungi, such as yeast, which may have growth-promoting abilities [61]. However, this possibility does not exclude the existence of a non-mycorrhizal relation between the AM fungi from the commercial inoculum and the plants.

Commercial AMF inoculum is considered to be at the needed standards for using them for the stimulations of plant development, but their effects depend on the soil and ecological context [62].

In order to claim that the effects in this study are related to the AM fungus, it was necessary to use a monoxenic spore culture of the AM fungus or to have a guarantee that any other microorganisms (other than the declared strain of AMF) are not present in the commercial product. However, our interest was from an applied perspective, and the costs of using monoxenic spore cultures would have been too high. According to our knowledge, no producer of commercial AMF inoculum in quantities relevant to field technologies can guarantee that other microorganisms are not present in the sequestering material besides the species of interest. Taking into consideration the dump material was sterilized, it is reasonable to cautiously assume that an effect of AMF cannot be excluded as a potential cause of the observed patterns.

It is advisable to first investigate the effectiveness of commercial microbial inoculum at a small scale before field application in order to reduce the risks of failure and the associated costs [63]. The field application of commercial inoculum may not always have desired effects, as demonstrated recently in a mining area [64].

### 4.2. Effects on Dependent Variables: Elements, Oxidative Stress, and Biomass

Uranium mining dumps can be a source of heavy metals accumulated in plants growing on their surface [65]. Although the concentrations of toxic elements in the mining dump substrate used in the experiment reported here were not large in absolute values, their large availability was demonstrated by the concentrations’ increase in plants compared to the reference soil. 

Acid rain may not always have an independent effect from the pollution with metals on plant growth [66]. In other cases, the phytotoxicity of metals in a mine tailing was controlled both by pH and heavy metals, leading to a patchy distribution of vegetation and a lower number of species in acid spots [67]. In our case, the extra acidification by watering with acid water did not increase the toxic elements’ concentrations in roots and aboveground parts but increased those of Ca and decreased those of K.

The behavior of calcium in mining dumps has rarely been studied. Ca is used for the neutralization of the acidity of dumps, but it is leached and transferred to vegetation so that the substrate returns in several years to the state before liming [68]. In the experiment reported here, there was a normal increase in Ca concentrations in both roots and aboveground parts due to acid water. As for potassium, there are few studies of K dynamics in post-mining soils [69], but many authors report that its availability to plants decreases with the soil pH dropping below neutral [70]. K is leached from the rhizosphere in the presence of clay minerals controlled by the low pH [71]. Depending on the soil characteristics, K availability can also decrease at pHs towards 8 [72].

In the experiment reported here, we did not find an increase in Al concentrations, usually associated with low pHs, probably due to the small effect of the acidification with water (decrease with less than 0.5 pH units of the dump material, Table S1). Still, this slight decrease diminished the readily available K pool, which converges with previous reports [73]. 

Mining dumps suffer losses of P by erosion which reduces its already low availability [74]. The decrease in P in plants from the normal soil to the dump material found in our experiment is something usual. The nonmycorrhizal phosphorus acquisition specific to lupine species [75] did not cope with the even lower decrease in pH by watering with acid water. It is on this background that the inoculation led, without roots’ colonization, to an increase in P in plants compared to the not inoculated dump material. Potential mechanisms underlying the effects of AMF will be discussed in detail in the next discussion’s subchapters. Different from our results, other authors demonstrated that AMF species could inoculate *L. albus* at rates between 13.3 and 30% but with no effects on the plant growth or on P and K concentrations [76].

Facilitating P uptake is more beneficial for plants under P-deficient and acid soil conditions [77]. While the effect of soil pH decrease on lupine performance has not been studied, as far as we know, the soil pH increase by liming is common. The pH increases from 4.7–5.3 to 6.3 decreasing the capacity of *L. angustifolius* to uptake P [78], but this pattern depends on the soil conditions and differs from one lupine species to another [79].

The interplay between the above-described changes in nutrient concentrations and toxic elements concentration in plants led to changes in plant biomass and oxidative stress variables. Such modifications are normal in plants grown in mining dump substrates and acid conditions and have been reported in the past by many authors. For instance, a pot experiment with *Rumex acetosa* exposed to acid rain demonstrated increased Cu uptake and consequent oxidative stress [80].

### 4.3. Research Directions

**Figure 5:**
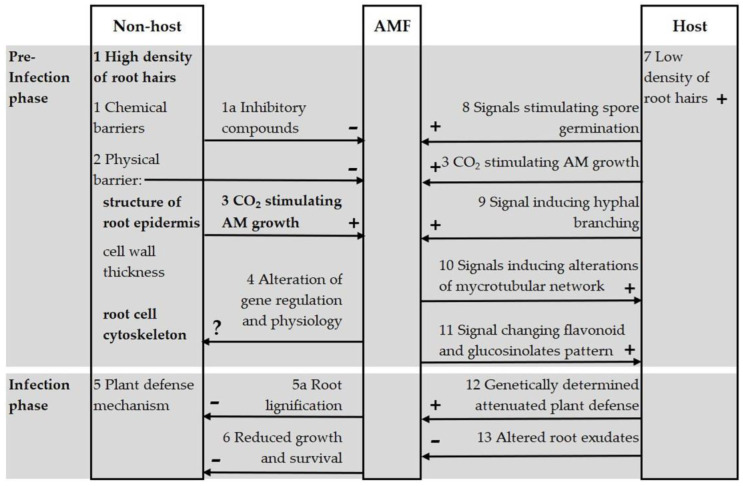
Interaction mechanisms of AMF with host and non-host plants.

If a species classified as non-host hypothetically had ecological relations (other than symbiotic) with AMF when the physiological adaptations of the non-host plant were no longer efficient, such as in acid mining dump conditions, this raises the questions of the potential mechanisms. In the Supplementary discussion, we review the literature [81,82,83,84,85,86,87,88,89,90,91,92,93,94,95,96,97,98,99,100,101,102,103,104,105,106] allowing a comparison between interaction mechanisms of AMF with host and non-host plants, summed up in (Figure 5).

From the point of view of applied science, there are two important issues to emphasize: that lupine is a suitable plant for remediation of acid metals polluted ecosystems, and that lupine is a good first plant when using crop rotation to accelerate secondary succession during remediation these systems [37]. *L. albus* was used for the common phytoremediation of soil contaminated with heavy metals and persistent organic pollutants [107]. Based on experimental evidence, *L. uncinatus* is recommended for the phytostabilization of soil contaminated with Cd [6]. Other authors found that white lupine grown in an acid soil (pH 2.5–5.5) accumulated Cd and As in the roots and highlighted the usefulness of this species for phytostabilization due to its beneficial effects on the soil properties, particularly an increase in soil pH and a decreased in Cd and As solubility [108].

*L. albus* was found appropriate for the phytoimmobilization of heavy metals contaminated soils [109]. *L. angustifolius* was found to spontaneously colonize an acidic area contaminated with heavy metals [110]. An insight into the potential of lupine species for remediation (phytostabilization or phytoextraction) can also be obtained from the agronomic literature. *L. luteus* used all P sources more efficiently than *L. angustifolius* to produce dry shoots and grain. An insight into the potential of lupine species for remediation (phytostabilization or phytoextraction) can also be obtained from the agronomic literature. For each amount of each P fertilizer source applied, the concentration of Cd in grain was always higher for *L. luteus* [111]. There are also differences between these two species in what concerns Zn uptake [112]. A comparison of *L. angustifolius* and *L. luteus* in Cd polluted soils showed that grain yield of narrow-leafed lupine was not significantly reduced by the addition of Cd to the soil, while this happened for yellow lupine [113].

In cropping systems involving non-mycorrhizal and mycorrhizal plants, or pre-cropping with N fixation and P extractors are possible strategies for the phytoremediation of heavy metals contaminated sites. From an agronomical perspective, independent but applicable to remediation projects, large amounts of N can be fixed by field-grown lupins, amounts far in excess of the quantities of N harvested in the seed [114]. There was evidence for the cycling of lupine root-derived N into soil microbial biomass and soluble organic N during lupine growth through the late vegetative stage [115]. The integration of P-efficient species into the cropping system could also improve the P acquisition of the main crop in acid soils by enhancing root colonization by mycorrhiza [116].

In a crop rotation experiment, the incorporation of lupine green manure stimulated microbial community growth and activity [117]. The use of lupine as a pioneer produced good results in terms of the revegetation of eroded areas, improvement of soil reclamation, and dune stabilization in different climatic conditions [118].

The results reported in this article suggest the possibility of using a commercial AMF inoculum with non-host lupine in acid-contaminated soils as a first crop before an AMF host species. In future work, one might check the patterns reported here on other, more contaminated mining dump materials at the same (pot) and larger scale (lysimeter). Another possibility is to use a second crop with a host plant without extra inoculation with AMF and look for eventual symbiosis from the initial inoculum provided to the lupine crop. 

## 5. Conclusions

*L. angustifolius*, a non-host species, benefited at the time scale of one month from the commercial AMF inoculation of a mining dump substrate, especially when watered with acid water. There was no mycorrhization under the experimental conditions, but the fresh weight of stems and leaves significantly increased due to inoculation (under acid water conditions). In both neutral and acidic water conditions, the P concentrations in roots and leaves significantly increased, and superoxide dismutase and peroxidase activities were significantly decreased due to the commercial AMF inoculum. The increase in P concentration in leaves was significantly correlated with the decrease in peroxidase activity. At the end of the experiment, the concentration of ammonium in the substrate was higher in the inoculated treatments than in the uninoculated ones.

As we did not use a monoxenic spore culture of the AM fungus or have assurance that any other microorganisms than the declared strain of AMF are not present in the commercial product, it is not certain that the observed effects are due to AMF.

In future work, a possibility is to study the microbiome of the AMF inoculum used in order to identify associated plant growth-promoting microorganisms. One might also check the patterns reported here on other, more contaminated soils at the same (pot) and larger scale (lysimeter). Another possibility could be to use a second crop with a host plant without extra inoculation with AMF and look for eventual symbiosis from the initial inoculum provided to the lupine crop.

## Figures and Tables

**Table 1:** Physicochemical variables measured for soil characterization. Averages are for eight pots with the same type of substrate. The symbol * shows that the average in D pots is significantly different than the average in R pots (*t*-test for independent groups). The symbol ^#^ shows that the average in DF pots is significantly different than the average in D pots (*t*-test).

Soil Variables	R		D		DF	
	Average	SD	Average	SD	Average	SD
Soil texture	Loamy sand		Sandy loam soil (10–30% loam)			
pH (H_2_O)	6.58 (composite)		4.88 (composite)			
N-NH_4_^+^ [mg kg^−1^]	38.7 (composite)		4.58	2.69	7.03	3.46
N-N0_3_^−^ [mg kg^−1^]	48.4 (composite)		0.27	0.12	0.30	0.16
LOI [%]	2.08	0.33	2.48 *	0.20	2.57	0.13
Elements [mg kg^−1^]						
Al	6908	265.1	11193 *	177.7	12,443 ^#^	93.06
As	13.12	1.29	19.36 *	1.26	20.37	1.64
Ba	35.15	2.21	92.79 *	3.10	95.94	5.69
Ca	1810	88.49	2021 *	152.0	1986	283.7
Cd	1.75	0.48	3.24 *	0.20	3.05	0.45
Co	4.06	0.42	15.17 *	0.49	14.77	0.38
Cr	14.74	0.54	32.42 *	2.53	30.81	1.16
Cu	21.36	1.30	46.89 *	0.75	46.02	1.30
Fe	7152	253.3	42272 *	732.9	41549	730.5
K	1042	64.51	1261 *	41.49	1417 ^#^	173.9
Mg	1395	51.29	3077 *	43.61	2959 ^#^	29.35
Mn	246.4	5.94	713.7 *	10.13	712.3	16.30
Mo	0.41	0.11	2.05 *	0.26	2.17	0.24
Ni	8.55	1.01	46.06 *	1.73	44.08 ^#^	1.48
P	376.8	10.01	489.5 *	12.98	491.4	7.16
Pb	27.91	8.38	24.27	7.47	20.62	4.38
Sr	21.61	0.75	18.94 *	1.05	19.35	1.06
Ti	159.1	9.18	410.3 *	9.49	416.8	16.33
V	16.70	0.49	40.55 *	0.86	41.11	1.02
Zn	93.15	26.29	77.56	4.94	60.75 ^#^	3.07

LOI = loss on ignition.

**Table 2:** Experimental treatments and their preparation.

Soil Type	Soil Code	Water Type	Number of Pots	Amount of Soil [g]	Amount of Clay Plus Fungi [g]
Reference	R	Neutral	4	355	0
	R	Acidic	4	355	0
Dump material without fungi	D	Neutral	4	355	0
	D	Acidic	4	355	0
Dump material with fungi	DF	Neutral	4	320	35
	DF	Acidic	4	320	35

**Table 3:** Ratios between the concentrations of elements in the soil of the experimental treatment at the end and at the start of the experiment. Only the ratios for statistically significant differences between means (one-way ANOVA) are presented. Fe and Ni concentrations did not change in any treatment.

Treatments	Al	As	Ca	Cr	Cu	K	Mg	Mn	P	Sr	Zn
R				0.95	0.26	0.85	0.89	0.95	0.93	0.92	0.26
RA					0.27	0.80	0.86	0.93	0.90	0.88	0.36
D			0.84		0.74	0.87	0.93		0.94		0.66
DA					0.73	0.86	0.92	0.96	0.92	0.90	0.72
DF	0.97				0.81		0.94		0.93		0.88
DFA	0.97	0.87			0.78		0.93		0.92		0.85

**Table 4:** Changes in the concentrations of several elements in the DF treatments compared with D treatments (*t*-test) at the start and the end of the experiment. The trends in the substrate due to the addition of expanded clay by the commercial inoculum at the start of the experiment were also found at the end of the experiment, except for Zn. The trends in roots and leaves did not follow those existing in the substrate at the start of the experiment.

	Start	End of the Experiment		
	Substrate	Substrate	Plants	
			Roots	Leaves
Al	Increase	Increase		
Cu		Increase		
K	Increase	Increase		
Mg	Decrease	Decrease		
Ni	Decrease	Decrease		
P			Increase	Increase
Sr		Increase	Increase	
Zn	Decrease		Increase	

## Data Availability

The raw data produced by the experiment reported in this article are available following the link provided in the Supplementary Materials.

## Supplementary Materials

The following supporting information can be downloaded at: https://www.mdpi.com/article/10.3390/plants12101983/s1. The raw data set; Figure S1: The experimental context of the bivariate experiment reported in this article and images of the vegetation chamber and lysimeters system with *Lupinus angustifolius*; Figure S2: Scattergram of peroxidase activity vs. phosphorus concentration in leaves of plants grown in the dump material, four treatments. Codes are as in Figure 4 of the article. One can notice the effect of inoculation with AMF (triangle symbols separating from round symbols). The corresponding negative correlation is statistically significant; Table S1: Root elemental concentrations in the experimental variants (average and standard deviations) and ANOVA results for each bivariate sub-set; Table S2: Leaves elemental variables in the experimental variants (average and standard deviations) and ANOVA results for each bivariate sub-set; Table S3: Stem and leaves biological variables in the experimental variants (average and standard deviations) and ANOVA results for each bivariate sub-set; Table S4: Root biological variables in the experimental variants (average and standard deviations) and ANOVA results for each bivariate sub-set; Table S5: Correlations between biological variables and elemental concentrations in roots and leaves; Table S6: Substrate variables in the experimental variants at the end of the experiment (average and standard deviations) and ANOVA results; Supplementary discussion.
