# Supplementary material for: A Commercial Arbuscular Mycorrhizal Inoculum Alleviated the Effects of Acid Water on Lupinus angustifolius Grown in a Sterilized Mining Dump
Source: Plants (Basel). 2023 May 15;12(10):1983. doi: 10.3390/plants12101983 (PMC10222887; doi:10.3390/plants12101983)
Supplement: Supplementary file 1 [file plants-12-01983-s001.zip › plants-2301140-supplementary.pdf]

Supplementary Materials:

The raw data set is available for download **here**.

Fig.S1 The experimental context of the bivariate experiment reported in this article and images of the vegetation chamber and lysimeters system with *Lupinus angustifolius*.

Table S1 Root elemental concentrations in the experimental variants (average and standard deviations) and ANOVA results for each bivariate sub-set.

Table S2 Leaves elemental variables in the experimental variants (average and standard deviations) and ANOVA results for each bivariate sub-set.

Table S3 Stem and leaves biological variables in the experimental variants (average and standard deviations) and ANOVA results for each bivariate sub-set.

Table S4 Root biological variables in the experimental variants (average and standard deviations) and ANOVA results for each bivariate sub-set.

Table S5 Correlations between biological variables and elemental concentrations in roots and leaves.

Fig.S2 Scattergram of peroxidase activity vs. phosphorus concentration in leaves of plants grown in the dump material, four treatments. Codes are as in Figure 4 of the article. One can notice the effect of inoculation with AMF (triangle symbols separating from round symbols). The corresponding negative correlation is statistically significant.

Table S6 Substrate variables in the experimental variants at the end of the experiment (average and standard deviations) and ANOVA results.

Supplementary discussion

Fig.S1 ***Left:*** The experimental context of the bivariate experiment reported in this article. Soil number 1 is the soil D reported in this manuscript. The treatments with acidic water were performed only in the case of soil number 1 = D soil, as described in the manuscript. Soils 2 and 3 were very contaminated with heavy metals and are the subject of another publication. The results for soils 2 and 3 will be reported with data from two experimental scales: pots and lysimeters. ***Right:*** images of the vegetation chamber and lysimeters system with *Lupinus angustifolius*.

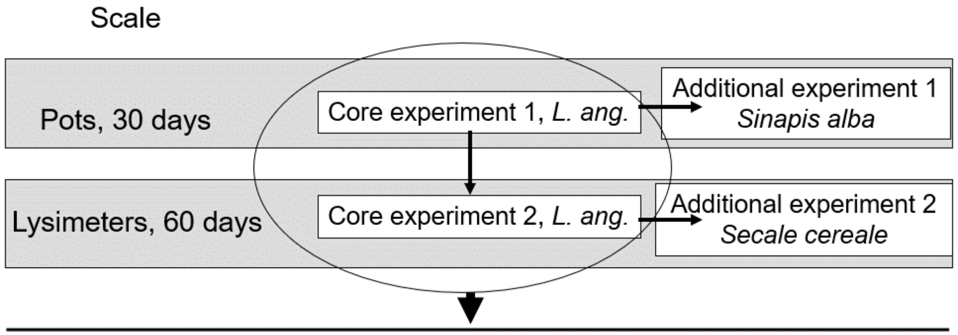

	30 days old plants, pots experiment						60 days old plants, lysimeters experiment					
	Biological parameters			Elements in plants			Biological parameters		Elements in plants		Elements in water	
	Soil 1	Soil 2	Soil 3	Soil 1	Soil 2	Soil 3	Soil 2	Soil 3	Soil 2	Soil 3	Soil 2	Soil 3
Not inoculated	x	x	x	x	x	x	x	x	x	x	x	x
Inoculated	x	x	x	x	x	x	x	x	x	x	x	x

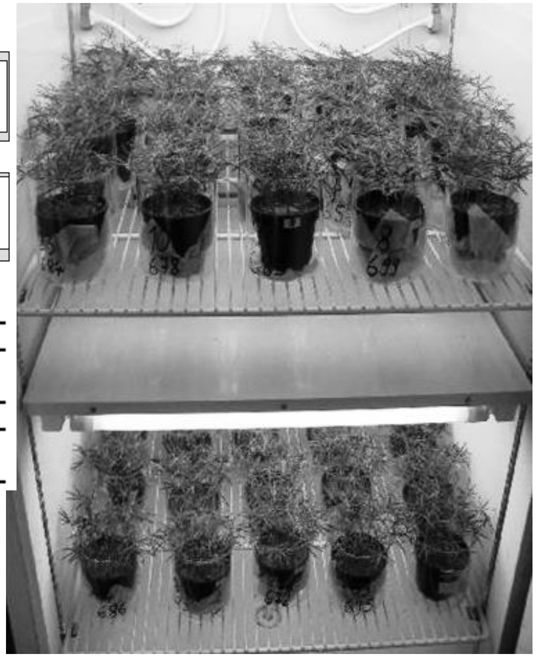

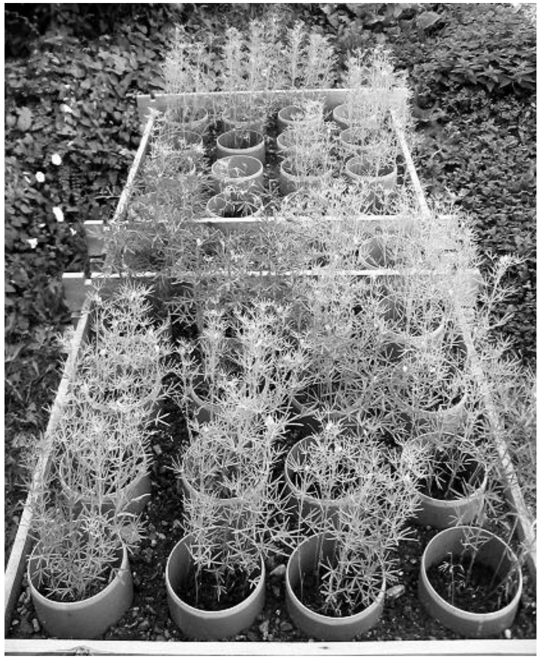

Table S1 Root elemental concentrations in the experimental treatments (average and standard deviations) and ANOVA results for each bivariate sub-set. NS = not significant.

Soil code	Inoculation Code	Watering code		Al	As	Ca	Cr	Cu	Fe	K	Mg	Mn	Ni	P	Sr	Zn
R = Reference - normal soil, D = mining dump material	0 = not inoculated, 1 = inoculated	0 = neutral water, 1 = acid water		[μg g^{-1}]	[μg g^{-1}]	[μg g^{-1}]	[μg g^{-1}]	[μg g^{-1}]	[μg g^{-1}]	[μg g^{-1}]	[μg g^{-1}]	[μg g^{-1}]	[μg g^{-1}]	[μg g^{-1}]	[μg g^{-1}]	[μg g^{-1}]
R	0	0	Average	3535	0.25	18874	105.44	12.26	4531	6673	13258	520.8	66.81	1396.0	91.14	58.01
			SD	*814*	*0.14*	*2020*	*52.13*	*8.89*	*1829*	*655*	*7268*	*179.9*	*84.66*	*150.2*	*41.28*	*8.84*
R	0	1	Average	2496	0.33	19768	98.10	10.34	2770	7616	9893	609.4	26.77	1502.1	115.34	67.34
			SD	*714*	*0.29*	*1167*	*39.30*	*4.16*	*467*	*394*	*490*	*85.7*	*8.49*	*96.8*	*11.36*	*9.60*
D	0	0	Average	2845	0.28	14819	68.73	25.92	7734	5895	20390	2453.6	165.76	1236.3	32.08	70.61
			SD	*232*	*0.07*	*855*	*12.07*	*4.64*	*1731*	*484*	*1969*	*328.6*	*14.75*	*77.7*	*1.97*	*7.68*
D	0	1	Average	2598	0.26	11198	53.42	24.69	6599	6733	17458	2099.3	145.55	1075.6	25.23	79.14
			SD	*1286*	*0.21*	*2209*	*38.18*	*6.66*	*2035*	*474*	*2904*	*540.7*	*29.69*	*196.1*	*2.12*	*15.13*
D	1	0	Average	3386	0.32	14260	55.85	42.64	8245	6446	18739	2364.4	178.73	1334.1	35.03	127.48
			SD	*546*	*0.15*	*1767*	*10.06*	*22.59*	*1569*	*360*	*1901*	*401.7*	*21.48*	*94.9*	*4.16*	*47.68*
D	1	1	Average	3433	0.20	13170	68.68	26.94	7643	7045	18634	2649.4	181.90	1281.9	30.29	78.70
			SD	*1497*	*0.17*	*2349*	*25.75*	*4.62*	*2963*	*835*	*2532*	*707.7*	*29.16*	*101.0*	*0.90*	*6.63*
bivariate ANOVA, p	Soil code			NS	NS	0.000	NS	0.001	0.001	NS	0.003	0.000	0.000	0.001	0.000	0.042
	Watering code			NS	NS	NS	NS	NS	NS	0.023	NS	NS	NS	NS	NS	NS
	Soil code*Watering code			NS	NS	0.019	NS	NS	NS	NS	NS	NS	NS	NS	NS	NS
bivariate ANOVA, p	Inoculation Code			NS	NS	NS	NS	NS	NS	NS	NS	NS	NS	0.033	0.009	0.047
	Watering code			NS	NS	0.028	NS	NS	NS	0.026	NS	NS	NS	NS	0.001	NS
	Inoculation Code*Watering code			NS	NS	NS	NS	NS	NS	NS	NS	NS	NS	NS	NS	0.044
Relative change (acid water - neutral water) *100/acid water (%)			soil	-29.39	35.56	4.74	-6.96	-15.63	-38.88	14.14	-25.38	-40.30	-59.93	7.60	26.55	16.07
			dump material	-8.69	-30.27	-24.44	-22.28	-4.73	-14.68	9.29	-14.38	-14.44	-12.19	-12.99	-21.34	12.07
			dump + AMF	1.37	-37.63	-7.64	22.96	-36.83	-7.31	14.21	-0.56	12.05	1.78	-3.92	-13.53	-38.26

Table S2 Leaves elemental variables in the experimental treatments (average and standard deviations) and ANOVA results for each bivariate sub-set. UDL = under the detection limit, NS = not significant.

Soil code	Inoculation Code	Watering code		Al	As	Ca	Cr	Cu	Fe	K	Mg	Mn	Ni	P	Sr	Zn
R = Reference - normal soil, D = mining dump material	0 = not inoculated, 1 = inoculated	0 = neutral water, 1 = acid water		[μg g^{-1}]	[μg g^{-1}]	[μg g^{-1}]	[μg g^{-1}]	[μg g^{-1}]	[μg g^{-1}]	[μg g^{-1}]	[μg g^{-1}]	[μg g^{-1}]	[μg g^{-1}]	[μg g^{-1}]	[μg g^{-1}]	[μg g^{-1}]
R	0	0	Average	190.13	UDL	9698	6.60	6.04	330.40	7787.5	3383.8	579.00	4.71	1666.3	60.86	46.11
			SD	*96.85*		*919*	*7.63*	*0.49*	*138.26*	*49.9*	*113.9*	*108.38*	*3.47*	*61.4*	*7.06*	*3.02*
R	0	1	Average	116.03	UDL	10860	3.59	5.48	290.25	6777.5	2797.9	690.63	2.12	1649.9	65.86	45.21
			SD	*24.15*		*2479*	*2.05*	*1.58*	*42.02*	*1395.6*	*463.3*	*132.36*	*0.69*	*353.0*	*16.08*	*9.24*
D	0	0	Average	250.28	UDL	12731	9.38	10.58	682.25	8032.5	5533.8	1490.13	18.13	1529.1	34.23	62.01
			SD	*147.12*		*880*	*6.41*	*3.38*	*366.61*	*983.3*	*2091.8*	*642.13*	*11.34*	*197.9*	*21.61*	*13.21*
D	0	1	Average	214.14	UDL	14215	9.88	11.56	581.38	6752.5	5680.0	2044.63	22.06	1527.3	24.85	60.91
			SD	*98.50*		*454*	*9.46*	*0.72*	*254.47*	*1449.7*	*747.1*	*297.45*	*3.55*	*58.9*	*1.20*	*8.65*
D	1	0	Average	192.29	UDL	13279	7.58	12.63	554.25	7410.0	5833.8	1924.00	21.53	1739.4	22.90	69.81
			SD	*79.29*		*1095*	*4.70*	*1.66*	*172.15*	*1353.2*	*614.5*	*129.84*	*2.26*	*106.2*	*2.47*	*5.40*
D	1	1	Average	248.70	UDL	16485	6.35	13.05	792.50	5805.0	5273.8	2319.38	23.33	1746.9	26.82	69.65
			SD	*71.57*		*1575*	*2.14*	*1.21*	*324.48*	*369.6*	*577.2*	*401.50*	*2.29*	*179.3*	*2.02*	*5.26*
bivariate ANOVA, p	Soil code			NS		0.001	NS	0.000	0.018	NS	0.001	0.000	0.000	NS	0.000	0.005
	Watering code			NS		NS	NS	NS	NS	NS	NS	NS	NS	NS	NS	NS
	Soil code*Watering code			NS		NS	NS	NS	NS	NS	NS	NS	NS	NS	NS	NS
bivariate ANOVA, p	Inoculation Code			NS		NS	NS	NS	NS	NS	NS	NS	NS	0.013	NS	NS
	Watering code			NS		0.002	NS	NS	NS	0.024	NS	0.040	NS	NS	NS	NS
	Inoculation Code*Watering code			NS		NS	NS	NS	NS	NS	NS	NS	NS	NS	NS	NS
Relative change (acid water - neutral water) *100/acid water (%)			soil	-38.97		11.99	-45.67	-9.20	-12.15	-12.97	-17.31	19.28	-55.00	-0.98	8.22	-1.95
			dump material	-14.44		11.65	5.32	9.34	-14.79	-15.94	2.64	37.21	21.70	0.43	-27.41	-1.77
			dump + AMF	29.34		29.00	-16.24	3.30	42.99	-21.66	-9.60	20.55	8.34	-0.12	17.11	-0.23

Table S3 Stem and leaves biological variables in the experimental treatments (average and standard deviations) and ANOVA results for each bivariate sub-set.

SOD = superoxide dismutase activity, POD = peroxidase activity, LP = lipids peroxidation, NS = not significant.

				Shoots			Leaves							
**Soil code**	**Inoculation Code**	**Watering code**		**Height of individuals (average, n = 5)**	**Fresh weight**	**Dry weight**	**Fresh weight**	**Dry weight**	**Protein**	**SOD**	**POD**	**LP**	**Chlorophyll**	**Carotenoids**
R =Reference-normal soil, D = mining dump material	0 = not innoculated, 1 = innoculated	0 = neutral water, 1 = acid water		[cm]	[g f.w.]	[g d.w.]	[g f.w.]	[g d.w.]	[μg g^{-1} d.w.]	[U mg^{-1} protein]	[μUnits mg^{-1} protein]	[μmol MDA g^{-1} d.w.]	[μg g^{-1} d.w.]	[μg g^{-1} d.w.]
R	0	0	Average	7.05	1.53	0.30	8.53	2.20	12457	174.06	2.03	0.31	14.53	0.49
			SD	*0.41*	*0.06*	*0.02*	*0.34*	*0.21*	*5284*	*76.91*	*1.61*	*0.04*	*2.60*	*0.07*
R	0	1	Average	7.00	1.43	0.28	7.42	1.74	8804	234.14	3.76	0.32	11.01	0.38
			SD	*0.31*	*0.10*	*0.02*	*0.82*	*0.28*	*1454*	*38.53*	*2.15*	*0.05*	*2.94*	*0.08*
D	0	0	Average	6.22	1.39	0.23	7.45	1.60	9041	225.68	5.56	0.37	9.78	0.37
			SD	*0.31*	*0.07*	*0.02*	*0.40*	*0.20*	*683*	*32.70*	*1.75*	*0.04*	*1.71*	*0.02*
D	0	1	Average	5.51	1.12	0.23	5.79	1.35	8718	272.73	4.30	0.41	8.10	0.32
			SD	*0.50*	*0.11*	*0.06*	*0.67*	*0.25*	*1126*	*23.32*	*1.50*	*0.03*	*2.12*	*0.02*
D	1	0	Average	6.18	1.33	0.23	7.53	1.71	12856	148.30	1.74	0.33	6.90	0.28
			SD	*0.16*	*0.09*	*0.03*	*0.07*	*0.23*	*1083*	*21.06*	*0.61*	*0.02*	*1.75*	*0.03*
D	1	1	Average	7.16	1.42	0.29	6.87	1.59	8815	227.07	2.63	0.38	7.90	0.31
			SD	*0.45*	*0.18*	*0.03*	*0.50*	*0.18*	*976*	*59.02*	*1.28*	*0.04*	*1.40*	*0.02*
bivariate ANOVA, p	Soil code			0.000	0.000	0.008	0.001	0.001	NS	NS	0.041	0.002	0.008	0.005
	Watering code			NS	0.001	NS	0.001	0.011	NS	0.043	NS	NS	NS	0.012
	Soil code*Watering code			NS	NS	NS	NS	NS	NS	NS	NS	NS	NS	NS
bivariate ANOVA, p	Inoculation Code			0.001	NS	NS	0.027	NS	0.002	0.006	0.002	NS	NS	0.002
	Watering code			NS	NS	NS	0.000	NS	0.001	0.005	NS	0.017	NS	NS
	Inoculation Code*Watering code			0.001	0.011	NS	NS	NS	0.003	NS	NS	NS	NS	0.005
Relative change (acid water - neutral water) *100/acid water (%)			soil	-0.64	-6.38	-8.07	-12.99	-20.77	-29.33	34.52	85.18	4.40	-24.23	-22.13
			dump material	-11.34	-19.64	-0.05	-22.20	-15.82	-3.58	20.85	-22.70	10.63	-17.18	-14.80
			dump + AMF	15.95	6.19	26.43	-8.80	-6.97	-31.43	53.12	51.55	16.89	14.52	10.82

Table S4 Root biological variables in the experimental treatments (average and standard deviations) and ANOVA results for each bivariate sub-set. SOD = superoxide dismutase activity, POD = peroxidase activity, LP = lipids peroxidation, NS = not significant.

Soil code	Inoculation Code	Watering code		Fresh weight	Dry weight	Protein	SOD	POD	LP
R = Reference - normal soil, D = mining dump material	0 = not inoculated, 1 = inoculated	0 = neutral water, 1 = acid water		[g f.w.]	[g d.w.]	[µg g^{-1} d.w.]	[U mg^{-1} protein]	[µUnits mg^{-1} protein]	[µmol MDA g^{-1} d.w.]
R	0	0	Average	21.91	1.91	2091	1439	77.00	1.05
			SD	*2.10*	*0.22*	*338*	*812*	*19.56*	*0.10*
R	0	1	Average	20.97	1.64	3567	1658	39.96	1.09
			SD	*1.65*	*0.15*	*1097*	*1555*	*27.95*	*0.16*
D	0	0	Average	16.37	1.40	2802	1590	57.46	1.08
			SD	*0.86*	*0.12*	*275*	*619*	*9.44*	*0.06*
D	0	1	Average	13.50	1.20	2026	1942	94.91	1.20
			SD	*2.18*	*0.12*	*747*	*2115*	*29.11*	*0.15*
D	1	0	Average	16.73	1.40	1584	1091	77.87	1.02
			SD	*1.05*	*0.03*	*218*	*283*	*23.28*	*0.08*
D	1	1	Average	14.93	1.22	1810	1623	91.66	1.13
			SD	*2.13*	*0.03*	*462*	*422*	*10.98*	*0.14*
bivariate ANOVA, p	Soil code			0.000	0.000	NS	NS	NS	NS
	Watering code			NS	0.013	NS	NS	NS	NS
	Soil code*Watering code			NS	NS	0.007	NS	0.007	NS
bivariate ANOVA, p	Inoculation Code			NS	NS	0.010	NS	NS	NS
	Watering code			0.016	0.001	NS	NS	0.025	NS
	Inoculation Code*Watering code			NS	NS	NS	NS	NS	NS
Relative change (acid water - neutral water) * 100/acid water (%)			soil	-4.29	-14.30	70.62	15.22	-48.10	4.23
			dump material	-17.53	-14.19	-27.69	22.12	65.19	10.89
			dump + AMF	-10.74	-12.58	14.29	48.81	17.70	11.53

Table S5 Correlations between biological variables and elemental concentrations in roots (up) and leaves (down).

Roots variabiles	Marked correlations are significant at p < .05000 N=24 (Casewise deletion of missing data)															
	Al	As	Ba	Ca	Cr	Cu	Fe	K	Mg	Mn	Mo	Ni	P	Sr	Ti	Zn
Fresh weight	.0198	.2145	.4571	.7642	.4998	-.3457	-.5507	.2158	-.4436	-.6636	.0476	-.5517	.5402	.7093	-.0331	-.2192
	p=.927	p=.314	p=.025	p=.000	p=.013	p=.098	p=.005	p=.311	p=.030	p=.000	p=.825	p=.005	p=.006	p=.000	p=.878	p=.303
Dry weight	.1389	.0994	.4913	.7405	.5202	-.4456	-.4976	-.0268	-.5289	-.7227	-.0815	-.6308	.5177	.7026	-.0179	-.2912
	p=.518	p=.644	p=.015	p=.000	p=.009	p=.029	p=.013	p=.901	p=.008	p=.000	p=.705	p=.001	p=.010	p=.000	p=.934	p=.167
Protein	-.0793	.3620	.1906	.3845	.1099	-.3487	-.2743	.2467	-.3632	-.3875	.0315	-.4615	.4598	.4683	.1537	-.1955
	p=.713	p=.082	p=.372	p=.064	p=.609	p=.095	p=.195	p=.245	p=.081	p=.061	p=.884	p=.023	p=.024	p=.021	p=.473	p=.360
SOD	-.1599	-.0767	-.0652	.1072	-.2768	-.1801	-.2722	-.1520	-.1159	.1660	.0782	-.0808	-.0201	.0902	-.0163	-.1181
	p=.455	p=.722	p=.762	p=.618	p=.190	p=.400	p=.198	p=.478	p=.590	p=.438	p=.717	p=.707	p=.926	p=.675	p=.940	p=.583
POD	.0998	-.3149	-.2546	-.3548	-.3025	-.0437	.1877	-.2441	.1850	.3145	-.0363	.2675	-.3601	-.3305	.0134	-.0477
	p=.643	p=.134	p=.230	p=.089	p=.151	p=.839	p=.380	p=.250	p=.387	p=.134	p=.866	p=.206	p=.084	p=.115	p=.951	p=.825
LP	.1482	-.0937	-.1163	-.3128	-.2875	-.1078	.1976	.0985	-.0386	.0785	.3234	-.0000	.1531	-.0950	.3024	.0090
	p=.490	p=.663	p=.588	p=.137	p=.173	p=.616	p=.355	p=.647	p=.858	p=.715	p=.123	p=1.00	p=.475	p=.659	p=.151	p=.967

Leaves variables	Marked correlations are significant at p < .05000 N=24 (Casewise deletion of missing data)															
	Al	As	Ba	Ca	Cr	Cu	Fe	K	Mg	Mn	Mo	Ni	P	Sr	Ti	Zn
Fresh weight	-.2239	--	.3775	-.5528	-.2785	-.4454	-.3923	.3951	-.3915	-.5740	-.6216	-.5326	.2247	.5160	-.2538	-.2926
	p=.293	p= ---	p=.069	p=.005	p=.188	p=.029	p=.058	p=.056	p=.059	p=.003	p=.001	p=.007	p=.291	p=.010	p=.231	p=.165
Protein	.2771	--	.2464	-.1512	.2797	-.0433	.0524	.2633	.0167	-.0579	-.2444	.0016	.1174	.0688	.0571	.0149
	p=.190	p= ---	p=.246	p=.481	p=.186	p=.841	p=.808	p=.214	p=.938	p=.788	p=.250	p=.994	p=.585	p=.749	p=.791	p=.945
SOD	-.2784	--	-.4269	.0832	-.1721	-.0226	-.1187	-.3355	.0012	.0232	.2906	.0213	-.3067	-.1356	-.1032	-.1133
	p=.188	p= ---	p=.037	p=.699	p=.421	p=.916	p=.581	p=.109	p=.996	p=.914	p=.168	p=.921	p=.145	p=.527	p=.631	p=.598
POD	-.1790	--	-.4231	-.1060	-.0492	-.1134	-.0555	-.0622	-.0016	-.0735	.1040	-.0033	-.5760	-.1797	-.1008	-.2248
	p=.403	p= ---	p=.039	p=.622	p=.819	p=.598	p=.797	p=.773	p=.994	p=.733	p=.629	p=.988	p=.003	p=.401	p=.639	p=.291
LP	.3358	--	-.2016	.5190	.2116	.5075	.4718	-.0750	.4821	.6179	.4932	.5517	-.1561	-.4866	.3225	.4258
	p=.109	p= ---	p=.345	p=.009	p=.321	p=.011	p=.020	p=.728	p=.017	p=.001	p=.014	p=.005	p=.466	p=.016	p=.124	p=.038
Chlorophyll	-.1281	--	.4985	-.5281	-.1307	-.7163	-.3863	.2189	-.6176	-.7320	-.5523	-.6732	-.0103	.7008	-.2701	-.7045
	p=.551	p= ---	p=.013	p=.008	p=.543	p=.000	p=.062	p=.304	p=.001	p=.000	p=.005	p=.000	p=.962	p=.000	p=.202	p=.000
Carotenoids	-.0091	--	.4464	-.6297	-.0121	-.7063	-.3267	.1807	-.5679	-.7008	-.5213	-.6142	-.1533	.6151	-.1796	-.6900
	p=.966	p= ---	p=.029	p=.001	p=.955	p=.000	p=.119	p=.398	p=.004	p=.000	p=.009	p=.001	p=.474	p=.001	p=.401	p=.000

Fig. S2. Scattergram of peroxidase activity vs. phosphorus concentration in leaves of plants grown in the dump material, four treatments. Codes are as in figure 4 of the main text. One can notice the effect of inoculation with AMF (triangle symbols separating from round symbols). The corresponding negative correlation is statistically significant.

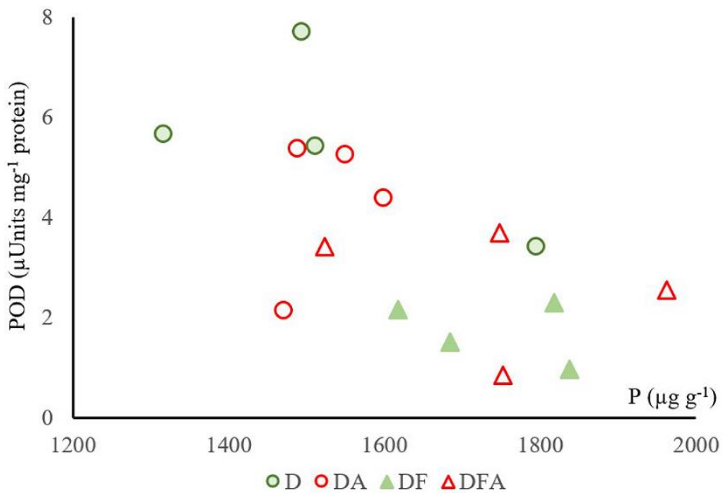

Table S6 Substrate variables in the experimental treatments at the end of the experiment (average and standard deviations) and ANOVA results for each bivariate sub-set. NS = not significant.

**Soil code**	**Inoculation Code**	**Watering code**		**pH**	**Loss on ignition**	**N-NO_3^-**	**N-NH_4^+**	**Al**	**As**	**Ca**	**Cr**	**Cu**	**Fe**	**K**	**Mg**	**Mn**	**Ni**	**P**	**Sr**	**Zn**
R =Reference - normal soil, D = mining dump material	R = not inoculated, D = inoculated	R = neutral water, 1 = acid water			[%]	[µg g^{-1}]	[µg g^{-1}]	[µg g^{-1}]	[µg g^{-1}]	[µg g^{-1}]	[µg g^{-1}]	[µg g^{-1}]	[µg g^{-1}]	[µg g^{-1}]	[µg g^{-1}]	[µg g^{-1}]	[µg g^{-1}]	[µg g^{-1}]	[µg g^{-1}]	[µg g^{-1}]
R	0	0	Average	6.13	2.42	11.82	33.23	6835	11.76	1676	13.67	5.70	6890	880	1242	232.4	7.22	355.0	19.61	30.50
			SD	*0.10*	*0.57*	*1.58*	*5.92*	*46*	*1.08*	*71*	*0.35*	*0.70*	*221*	*28*	*42*	*5.6*	*0.97*	*13.0*	*0.67*	*5.83*
R	0	1	Average	5.97	2.29	19.49	42.44	6482	12.01	1660	14.50	5.55	6851	832	1205	232.5	8.67	336.4	19.27	25.30
			SD	*0.19*	*0.06*	*7.50*	*5.68*	*333*	*1.56*	*99*	*0.54*	*0.50*	*447*	*46*	*80*	*8.0*	*0.42*	*26.4*	*1.09*	*2.53*
D	0	0	Average	4.84	2.72	16.39	30.65	11063	16.89	1736	30.70	34.60	41924	1105	2870	697.1	45.17	464.2	17.29	54.02
			SD	*0.51*	*0.22*	*1.77*	*2.40*	*89*	*2.02*	*70*	*1.26*	*1.15*	*1003*	*103*	*21*	*19.4*	*1.37*	*3.5*	*1.14*	*3.87*
D	0	1	Average	4.37	2.83	25.77	33.77	11099	18.04	1804	32.18	34.28	41384	1081	2831	686.7	45.70	446.5	16.79	53.32
			SD	*0.03*	*0.12*	*4.47*	*4.88*	*129*	*0.49*	*193*	*3.94*	*1.36*	*1203*	*78*	*86*	*15.1*	*1.63*	*18.8*	*0.91*	*1.86*
D	1	0	Average	4.83	2.81	19.04	37.56	12065	18.65	1898	29.09	36.91	41554	1152	2763	697.7	42.44	457.9	18.02	55.76
			SD	*0.05*	*0.08*	*3.61*	*2.48*	*100*	*0.57*	*364*	*0.87*	*0.13*	*657*	*133*	*28*	*15.4*	*0.81*	*6.9*	*0.74*	*1.60*
D1	1	1	Average	4.46	2.87	20.81	39.01	12100	18.18	1816	30.92	36.12	41014	1290	2757	690.5	43.94	451.5	18.93	49.14
			SD	*0.07*	*0.07*	*5.15*	*5.55*	*143*	*0.14*	*222*	*1.10*	*1.20*	*810*	*57*	*55*	*16.0*	*1.76*	*8.2*	*1.30*	*0.62*
bivariate ANOVA, p	Soil code			0.000	0.019	0.034	0.041	0.000	0.000	NS	0.000	0.000	0.000	0.000	0.000	0.000	0.000	0.000	0.000	0.000
	Watering code			0.043	NS	0.003	0.028	NS	NS	NS	NS	NS	NS	NS	NS	NS	NS	NS	NS	NS
	Soil code*Watering code			NS	NS	NS	NS	NS	NS	NS	NS	NS	NS	NS	NS	NS	NS	NS	NS	NS
bivariate ANOVA, p	Inoculation Code			NS	NS	NS	0.012	0.000	NS	NS	NS	0.002	NS	0.022	0.006	NS	0.009	NS	0.018	NS
	Watering code			0.007	NS	0.016	NS	NS	NS	NS	NS	NS	NS	NS	NS	NS	NS	0.048	NS	0.008
	Inoculation Code*Watering code			NS	NS	NS	NS	NS	NS	NS	NS	NS	NS	NS	NS	NS	NS	NS	NS	0.025

Supplementary discussion

From an ecological and evolutionary standpoint, there are 'continuums of associations' in the development of saprophytic fungi of the rhizosphere to mutualistic mycorrhizal fungus [81]. Every interaction depends on the dispositions and developmental stages of the plant and fungi partners as well as on environmental factors [81]. It became clear that there are no neutral interactions between fungal endophytes and plants but a balance of antagonism [5]. These authors also point out the greater phenotypic plasticity of endophytes compared to pathogenic fungi. The position of any AMF species along the mutualism to parasitism continuum is a complex function of the involved species and the local environmental conditions [82].

The ecological relationships between AMF and their host plants are known to range from mutualism (++) to parasitism (+ −), depending on the plant genotype, developmental phase, and environmental conditions [83]. The existing research concerning the ecological relationships between AMF and *non-host* plants is more limited. The research focused mainly on the effects of P availability on the non-host status of plants, with the underlying hypothesis that at low P availability, there will be a switch between the host and non-host character of the plant [84]. It is known that gene regulation and the physiology of the non-host plant can be altered by the presence of AMF [85]. Still, we lack knowledge concerning the control of environmental conditions on this influence.

Exposing plants to low pH and high metal concentrations might be favorable to an infection by the AMF It cannot be predicted based on the literature if this interaction would be beneficial or not to the plant, because fungal endophytes are highly plastic and adaptable to local conditions, the net outcome of the interaction being the result of a balance of antagonism [5]. But such an interaction might be beneficial to the plant, considering that the normal P acquisition mechanism of the selected model plant (lupine) is less efficient in very acid soils (pH 3-4). The plant exudates cannot further reduce the pH of the very acid soil, increasing it due to the pK of the organic acids [86]. The density of the hair roots responsible for P acquisition in non-host plants [87] is lowered by high soluble Al concentration specific to acid soils. Using or not the opportunity associated with AMF infection would depend on the genetic and physiological peculiarities of the plant. It would be reflected by P concentration in plant tissues and eventually biomass increase compared to uninoculated treatments Root infection in such root disruptive conditions does not need to be a true mycorrhizal symbiosis, with a production of spores. A limitation of the fungi-plant relationship to a peculiar pre-mycorrhizal phase is possible in principle, as well. Considering that the non-host lupine was reported to show fungal endophytes (other than AMF) in roots [81], it is theoretically possible that a symbiosis between AMF and non-host plants under root disruptive environmental conditions to occur without true mycorrhization.

In a screening of the non-host *Lupinus* genera [2] no arbuscular formation was observed in any of the 37 *Lupinus* species. But the growth of external hyphae was observed in thirty-three species of *Lupinus*, vesicles were observed in two species, and internal hyphae were observed in eight species [2]. Arbuscules are linked to misdiagnosis since they are used less often than vesicles to recognize associations in roots and apparently occur sporadically in non-mycorrhizal plants [88]. This may be a reason why non-host species like *Lupinus* sp. have been sometimes reported as infected with AMF [84].

The sporadically reported physiological influence of AMF inoculation on Lupinus species is not necessarily incompatible with the non-host character of Lupinus, because of the possible presence of extraradical hyphae, vesicles, and even intraradical hyphae. AMF develops around dead *Lupinus albus* roots forming hyphal "swellings" [89] which should not be confused with true "appresoria". Even though the AMF would not complete their life cycle, as they are obligate symbionts [90] their hyphae can leave long enough in the absence of host plants (four months after germination [91] in order to develop significant ecological relationships with the non-host and at the same time preserving their capacity to colonize host plants. Extraradical structures of AMF can survive the winter in the soil at their site of synthesis. Reductions in soil densities of spores in autumn, and of hyphae over winter, did not affect the capacity of the extraradical mycorrhizal system to colonize plants swiftly in the spring, even after the elimination of any potential contribution from the part of the mycorrhizae inside the roots [92].

Lupin was reported to show fungal endophytes in roots [81]. More specifically, the occurrence and value of AMF on lupins has been neglected except for brief reports for *L. luteus* (Asai 1948 and Maeda 1954 cited by [84]; Asai 1948 reported a significant increase in dry weight resulting from the formation of mycorrhiza but this has not been confirmed). *L. angustifolius*, *L. consentinii,* and *L. luteus* were weakly infected (< 10% of root length) with vesicular-arbuscular endophytes and hence he considered mycorrhizas as not having a value in their P uptake on nutritionally poor soil [84]. He reports that the infection was reduced further when soil moisture was high and by small additions of P to the soil. The P nutrition improvement could be the result of extracellular enzymes produced by the extraradical hyphae. This mechanism is concluded to be insignificant from the low quantitative contribution extracellular hyphae of AMF give to the total phosphatase activity in the soil, and from estimations of which processes that may be rate limiting in organic P mineralization [93]. On the other hand, P solubilization might be improved by the interaction of AMF with bacteria. In the nitrate-containing medium, *G. intraradices* external mycelium as well as three bacterial species studied [94] were rather inefficient P solubilizers, when growing individually. However, when *G. intraradices* external mycelium interacted with either *P. aeruginosa* or *P. putida*, the levels of soluble P in the medium significantly increased [94]. Still, another mechanism of AMF extraradical hyphae action on Lupinus is the interaction with plant growth-promoting rhizobacteria [95]. Extracellular material of bacterial origin containing cellulose produced around the attached bacteria may mediate fungal/bacterial interactions [96]. This can hold also for the colonization of *Lupinus* sp. if the AMF forms vesicles and internal hyphae.

The effect of *Lupinus* on AMF was evaluated especially in order to characterize the mycorrhiza inhibition mechanism. Generally, it was found that non-mycorrhizal plants, depending on the species, can stimulate the growth of fungi through rhizosphere effects, inhibit AMF growth, or have no effect on AMF growth [97]. Both spore germination and early hyphal extension were stimulated by the presence of all root systems irrespective of mycotrophic status, compared with the controls (no roots) [4]. However, roots from mycotrophic species supported significantly greater hyphal lengths after 3 and 4 weeks than any of the non-mycotrophic species [4]. The fact that all of the species examined here were capable of stimulating AM fungal spore germination and early hyphal growth indicates that the stimulatory signal(s) responsible for this first step in AM fungal development from resting spores is relatively nonspecific. It appeared that the mycotrophic species' roots produced spatial information that was perceived by the fungus while the non-mycotrophic roots did not [4].

The exudates produced by cluster-rooted lupin in large quantities; as well as by other non-host plants; play an important role in the interaction with AMF; either by including chemical signals regulating the interaction or simply as an organic carbon source allowing the development of AMF as saprophytes around the roots. It was suggested that the barriers to mycorrhizal infection in 'non-hosts' are intrinsic and more probably related to characteristics of the root cortex or epidermis than to any infection-inhibiting factors that might be released in root exudates [98]. Root exudates of two AMF non-host plants (mustard and sugar beet) significantly reduced root colonization in cucumber plants; whereas no such effect was observed when root exudates of the AM non-host plant *L. albus* were applied [99]. *L. albus* seems to be atypical of the genus *Lupinus*. Whereas compounds released by roots of *L. albus* affect neither spore germination [1] nor asymbiotic hyphal growth; compounds released by roots of other *Lupinus* species such as *L. luteus*; *L. cosentini* and *L. aridus* clearly inhibited asymbiotic hyphal growth (; in contrast with the infection of *L. luteus* and *L. cosentini* reported by [84]. On the other hand; L. albus was inoculated between 13 and 30% [76] in their experimental conditions

The intensive exudation by the roots of *Lupinus* sp. is an adaptation for P mobilization from soil, besides the fact that, like other legumes, they can fix atmospheric nitrogen [100]. The mechanisms of P mobilization are linked to organic acids and phospholipid surfactants production. Lecithin (a phospholipid surfactant) could be exuded by lupin into the rhizosphere soil volume, decreasing soil water content and hydraulic conductivity at any given soil water potential, and decreasing phosphate adsorption to soil particles [101]. Relatively more important, considerable amounts of carboxylates are released in response to P deficiency, especially in cluster-rooted plants like lupin [102, 86]. In white and blue lupin, the carboxylate efflux, mainly citrate and malate, was by a factor of 10-100 higher than in ryegrass [103]. At neutral pH-s the organic acids released by the

cluster roots are a source of rhizosphere acidification. The decrease in soil pH may be constrained to the rhizosphere and not measurable in the bulk soil [104]. If the mechanism of P mobilization is mostly linked to a decrease in soil pH, then one could expect that the release of organic exudates is less efficient in very acid soils, and AMF might play a complementary role.

The organic exudates increase also the solubility of metals such as Fe, Al, and Zn [102]. During organic acid anion exudation bursts, metals in the rhizosphere of cluster roots were strongly mobilized [105]. The concentrations of dissolved organic carbon derived from soil organic matter increased parallel to organic acid anions. Speciation calculations revealed that, during exudation, Al, Ca, Mn, and Zn in the cluster root rhizosphere were mainly bound with citrate, while Cu and Pb were always strongly bound to soil-derived dissolved organic matter. Their results indicated that cluster root exudation led on one hand to direct mobilization and complexation of metals like Al, Fe, and Zn by citrate and on the other hand to the mobilization of soil organic matter which complexes and solubilizes Cu and Pb [106]. One could expect that this solubilization of metals will affect not only their uptake by lupine [106] but also the oxidative stress parameters [39].

Based on the above elements it is reasonable to accept that the soil inoculation with *Glomus intraradices* might have had a beneficial effect on the development of *L. angustifolius* (in terms of biomass increase and oxidative stress decrease) with the decrease of the contaminated soil pH by acid water.
